# Laboratory selection for an accelerated mosquito sexual development rate

**DOI:** 10.1186/1475-2875-10-135

**Published:** 2011-05-20

**Authors:** Clelia F Oliva, Mark Q Benedict, Guy Lempérière, Jérémie Gilles

**Affiliations:** 1 Insect Pest Control Laboratory, Joint FAO/IAEA Programme of Nuclear Techniques in Food and Agriculture, International Atomic Energy Agency Laboratories, A-2444 Seibersdorf, Austria; 2 MIVEGEC, IRD, Montpellier, France; 3 Private Consultant, Mosquito Hunters; 4 Centre de Recherche et de Veille dans l'Océan Indien, Sainte-Clotilde, Réunion

## Abstract

**Background:**

Separating males and females at the early adult stage did not ensure the virginity of females of *Anopheles arabiensis *(Dongola laboratory strain), whereas two years earlier this method had been successful. In most mosquito species, newly emerged males and females are not able to mate successfully. For anopheline species, a period of 24 h post-emergence is generally required for the completion of sexual maturation, which in males includes a 180° rotation of the genitalia. In this study, the possibility of an unusually shortened sexual maturity period in the laboratory-reared colony was investigated.

**Methods:**

The effect of two different sex-separation methods on the virginity of females was tested: females separated as pupae or less than 16 h post-emergence were mated with males subjected to various doses of radiation. T-tests were performed to compare the two sex-separation methods. The rate of genitalia rotation was compared for laboratory-reared and wild males collected as pupae in Dongola, Sudan, and analysed by Z-tests. Spermatheca dissections were performed on females mated with laboratory-reared males to determine their insemination status.

**Results:**

When the sex-separation was performed when adults were less than 16 h post-emergence, expected sterility was never reached for females mated with radio-sterilized males. Expected sterility was accomplished only when sexes were separated at the pupal stage. Observation of genitalia rotation showed that some males from the laboratory strain Dongola were able to successfully mate only 11 h after emergence and 42% of the males had already completed rotation. A small proportion of the same age females were inseminated. Wild males showed a much slower genitalia rotation rate. At 17 h post-emergence, 96% of the laboratory-reared males had completed genitalia rotation whereas none of the wild males had.

**Conclusion:**

This colony has been cultured in the laboratory for over one hundred generations, and now has accelerated sexual maturation when compared with the wild strain. This outcome demonstrates the kinds of selection that can be expected during insect colonization and maintenance, particularly when generations are non-overlapping and similar-age males must compete for mates.

## Background

Malaria is the most important insect-transmitted disease with half of the world's population at risk of disease and mortality. In 2008, malaria led to nearly 900,000 deaths [1]. The high impacts on human health and on countries' economies have motivated campaigns for the eradication of the disease through methods controlling either the parasite *Plasmodium *spp. or its vectors. *Anopheles arabiensis *is one of the major African vectors of malaria. A feasibility study of using the sterile insect technique (SIT) for *An. arabiensis*, as part of an area-wide integrated pest management project [2] for population suppression, is currently being conducted in Northern Sudan and in Réunion [3]. SIT is based on the release of large numbers of sexually sterile males, which would mate with wild females and transfer their sterile spermatozoids for the fertilization of the eggs. If the sterile males successfully compete for mates, the wild population size will progressively diminish. A lower probability of contact between the vector and humans is, therefore, expected, and as a result pathogen transmission and disease incidence will decrease. Sexual sterilization can be accomplished by male mosquito exposure to ionizing radiation, resulting in random dominant lethal mutations in the germinal cells that cause the death of the developing embryos after fertilization.

Part of the research work for the implementation of SIT requires mating of radio-sterilized males with virgin females so as to identify the effect of irradiation on those males. Until 2 years earlier, the sex-separation method routinely used in this laboratory for *An. arabiensis *Dongola, consisted of separating females from males as adults, less than 18 h after emergence [4,5]. This procedure consistently ensured virginity of the females. However, recent experiments suggested the occurrence of early matings between males and females separated from one another between 12 to 16 h after their emergence.

Like many Diptera species, male mosquitoes are not immediately sexually mature after emergence: maturation includes a permanent 180° rotation of their genitalia [6]. The male mosquito genitalia consist of the 8^th ^to 10^th ^abdominal segments. Claspers tipped with claws enable the male to grasp the female for copulation and are located on segment 10^th ^[7]. When males emerge these claws are rotated dorsally, which prevents them from copulating until the rotation occurs. Rotation is driven by two sets of opposed and crossed muscles [8] and can happen equally frequently either clockwise or counter-clockwise [8,9]. The time to complete this event is species-specific. Aedine species show a great variation: *Aedes iriomotensis*, *Aedes albopictus*, *Aedes atriisimilis *were shown to rotate 180° respectively around 12, 22 and 40 hours after emergence [10]; 18 to 24 h were required for *Aedes aegypti *[8]; 30 h for *Aedes taeniorhynchus *[11] and nearly 4 days for *Aedes provocans *[12]. *Culex tritaeniorhynchus *and *Culex quinquefasciatus *completed the rotation in 19 h [13,14]. Finally, the species *Culiseta inornata *needed only 6 to 12 h [7]. The rate of genitalia rotation for anopheline mosquitoes is not yet reported. Only observations of the insemination status demonstrated that at least 24 h post-emergence are required for mating in *An. arabiensis *and *Anopheles gambiae s.s. *[15] as well as for *Anopheles stephensi *[16]. Besides the requirement for male maturation, females of most mosquito species are unreceptive during the first 30-60 h after emergence; although they may allow copulation, they will not become inseminated [17]. Mahmood and Reisen [16] showed that females of *An. stephensi *reached sexual maturity by the 2^nd ^night of life, though a very low proportion of females were inseminated by older males less than 12 h after their emergence.

In order to investigate the issue of sexual maturity in this laboratory colony, experiments were performed to determine the rate of sexual maturation over time in the current laboratory colony of *An. arabiensis *Dongola. This was compared with wild specimens collected directly in the field. The forces for laboratory selection due to the stock-keeping method and the possible consequences of this outcome on insects rearing and research are discussed.

## Methods

### Mosquito stock and rearing

#### Laboratory colony

All experiments used the Dongola strain of *An. arabiensis *[18] colonized in 2004 from specimens collected near the village of Dongola in Northern State, Sudan. The Dongola colony has been reared in the Insect Pest Control Laboratories, Joint FAO/IAEA Division of Nuclear Techniques in Food and Agriculture, since that time. Approximately 18 generations of *An. arabiensis *can be reared per year as the development of one generation takes around 20 days from egg hatching to reproductive adult stage. Therefore, this strain has been maintained for approximately 125 generations in discrete generations. The Dongola strain was reared in a climate-controlled room maintained at a temperature of 27 ± 1°C and 60 ± 10% relative humidity. The light regime was LD 12:12 h photoperiod, including dusk (1 h) and dawn (1 h). The same environmental conditions were used for all laboratory experiments. Larvae were reared in plastic trays (40 × 29 × 8 cm) at a density of approximately 500 first instar larvae (L1) per tray that contained ± 1.5 litre of deionised water and fed a diet of finely ground (224 μ-sieved) Koi Floating Blend^® ^(Aquaricare^®^, New York, USA). Pupae were collected and placed in small plastic cups inside a fresh adult cage for emergence. Adults were kept in standard 30 × 30 × 30 cm insect cages (Megaview Science Education Services Co, Ltd, Taiwan) and continuously supplied with 10% [w/v] sucrose solution with 0.2% methylparaben [19] Females were blood-fed weekly on defibrinated bovine blood. Gravid females were allowed to oviposit in plastic cups with black lining containing a wet sponge over which a filter paper was placed.

#### Wild Dongola strain

Pupae were collected along the Nile River bank in 2010 (Dongola, Northern State, Sudan) and allowed to emerge in laboratory cages. Room temperature was ca 40°C, because no air-cooling system was available.

#### Experimental set up

##### Effect of male-female separation methods on virginity

In the first group, females of *An. arabiensis *Dongola (generation F_118_) were separated from males at the adult stage, 12 to 16 h post-emergence, similarly to the method of Helinski & Knols [4]. A second group consisted of females that were isolated from males at the pupal stage to ensure their virginity. Sex-separation at the pupal stage was based on observation of the genitalia under a binocular microscope (MR4, 2009). Those two groups of females were mated with males (1:1) subjected to various sterilizing doses of radiation. Male pupae were collected from trays within a 4 h interval. Males were exposed to gamma rays emitted by a Cobalt-60 source (Gammacell220, MDS Nordion, Ottawa, Canada) with a dose rate of ca 9 Gy/min. Doses of 0, 40, 70 or 120 Gray (Gy) were applied 18-22 h after pupation. Pupae were placed on a wet filter paper at the centre of the chamber. A dosimetry system was used to measure the dose received by the lot based on Gafchromic^® ^dosimeter film HD-810 (International Specialty Products, NJ, USA); three dosimeters were included with each lot of insects and read after irradiation with a Radiachromic^® ^reader (Far West Technology, Inc., California, USA). Each treatment consisted of two replicates of 100 males and 100 females for the first group, and 50 males and 50 females for the second group. Females were blood-fed on a membrane with human blood collected in an anticoagulant tube. They were then placed in tubes for individual oviposition. Hatch rates of each family (*i.e*. progeny of one female) were recorded.

##### Observation of male genitalia rotation

**Figure 1:**
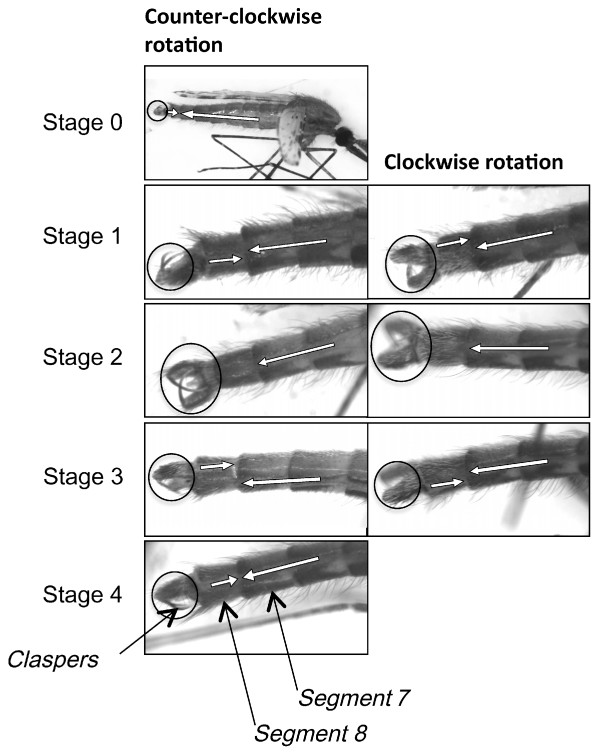
**Stages of genitalia rotation for *An. arabiensis***. Clockwise or counter-clockwise rotation, when the specimen is observed on its right side. Arrows indicate the position of pleuron 7 and 8.

The degree of genitalia rotation was recorded for males of different ages using the following divisional markers to categorize five stages of the rotation of the male genitalia [11,14], stage 0: no rotation (pleura of segments 7 and 8 continuous); stage 1: ≤45° rotation; stage 2: >45° - ≤90° rotation (basistyles perpendicular to the pleuron of segment 7); stage 3: >90° - ≤135° rotation and stage 4: >135° to complete rotation of 180° (pleura of segments 7 and 8 once more continuous, Figure 1). In all individuals that had completed rotation, the pleural line of the 7th and 8th segments of the genitalia were perfectly aligned only on one side but slightly shifted on the opposite side depending on whether rotation occurred clockwise or counter-clockwise. Thus, the genitalia had to be observed from both sides to allow determination of the final rotation stage: when claspers were ventrally rotated and the pleural lines were aligned on one side, the male was recorded as fully rotated. Such jagged lines have previously been reported and are due to the elasticity of the inter-segmental membranes [11]. According to the side that presented a perfect alignment, the rotational direction (clockwise or counter-clockwise) could be determined.

Emergence of all males spanned ca 2 h, all the males from one cage were removed at different time and frozen for later examination of the genitalia rotation stage. For the laboratory strain, these males were 0, 2.5, 3.5, 4.5, 5.5, 11, 12.5, 14, 15.5, 17 and 19.5 h, and 1 week old. Males aged from 2.5-5.5 h were obtained from a batch of pupae whose emergence was delayed to the morning by cooling the pupae at 15°C overnight, as a cooling treatment reduces the metabolic rate of pupae [5,20]. Concerning the wild males collected as pupae in the field, the degree of genitalia rotation was recorded only for 5 different ages due to a low number of individuals available: 0, 5.5, 13, 17 and 23 h.

##### Female insemination by laboratory reared males

Newly formed pupae of the laboratory reared Dongola strain (F_126_) were collected within a 4 h interval and sexed manually under a microscope. 65 male pupae and 55 female pupae were placed in each of 6 cages. Females were removed from the cages after a cohabitation period with the males of 11, 12.5, 14, 15.5, 17 and 19.5 h. In each cage a sample of five females was dissected and the spermatheca was examined for insemination, the remaining females were blood-fed and allowed to oviposit *en masse *in an egg cup. A batch of 40 female pupae was kept in a cage without any males as a control. They were blood-fed after seven days and an egg cup was introduced for eventual oviposition.

##### Statistical analysis

Normality and homoscedasticity of egg hatch data were examined using Shapiro and Bartlett tests. ANOVA was performed to test differences within one group between the mean egg hatch rates for each radiation treatment. T-tests (with Welch correction) were used to compare the mean egg hatch rates between the two groups of females and to compare the mean rotation stages between wild and laboratory reared males for a given time. Chi-squared tests (with Yates correction) were used to compare the proportions of wild and laboratory reared males for a given rotation stage and time. For all tests, the alpha level was *P <*0.05. Statistical analyses were performed using Microsoft Excel (Microsoft, USA) and the open source package R [21].

## Results

### Effects of male-female separation methods on virginity

**Figure 2:**
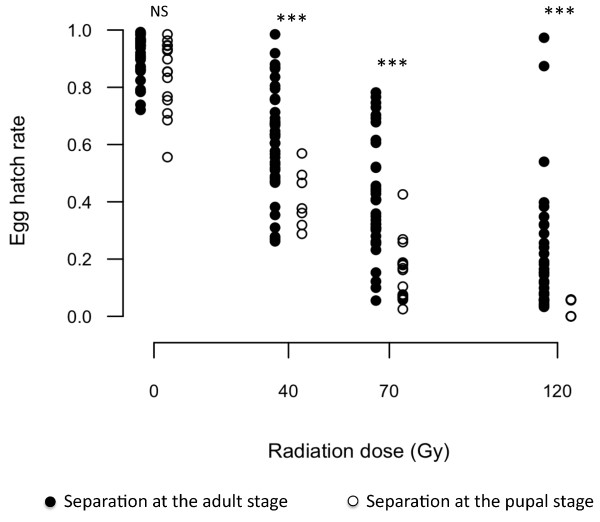
**Distribution of the egg hatch rates after mating with irradiated males**. Individual egg hatch rates for females separated as adults (black dots) or as pupae (circles) and mated with males irradiated at different doses. NS indicates no significant difference and *** indicates a significant difference (*P *< 0.001) between the two groups of females.

In the group where females were separated from males <16 h post-emergence at the adult stage, the mean egg hatch rate in the control was 91.0 ± 1.2% (mean ± sem); no reduction of fertility was visible among the irradiated treatments and full sterility was never reached (Figure 2). The mean fertility was significantly different between 70 and 120 Gy (F_4 _= 63.318, *P *< 0.001) but was still as high as 41.7 ± 3.3% and 21.2 ± 3.8% (mean ± sem) respectively. The distribution of individual egg hatch rates for the various radiation doses was similar and ranged over ca 80% indicating a high number of intermediate hatch rates (*i.e*. all values ranging between the expected sterility value and the control value). However this distribution was different in the group where females were separated from males at the pupal stage, and the intermediate hatch rates described earlier were no longer observed. In this group, results were in accord with the sterility curve obtained by Helinski *et al *[22]. Control mean fertility was 84.1 ± 3.2% (mean ± sem), and a proportional reduction was observed up to a dose of 120 Gy, where fertility was close to zero. Statistical comparisons between the two groups of females showed high significant differences between treatments where males were irradiated at 40 (t_14.5_= 4.16, *P *< 0.001), 70 (t_48.7 _= 6.21, *P *< 0.001) or 120 Gy (t_37.8 _= 4.59, *P *= 4.768e-05).

### Observation of male genitalia rotation and female insemination

**Figure 3:**
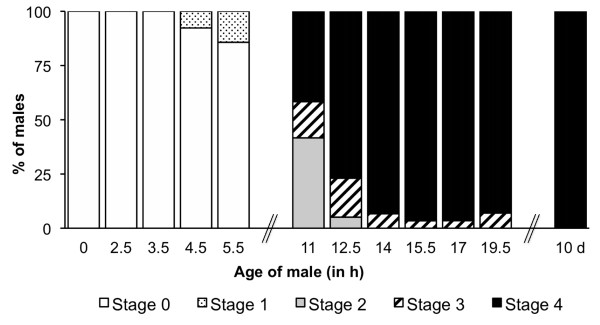
**Genitalia rotation over time**. Percentage of laboratory-reared males with the terminalia rotated in the different stages as a function of age.

All the 0-3.5 h old laboratory-reared males had non-rotated genitalia; rotation was evident at 4.5 h after emergence at which time only very few individuals had reached stage 1 (Figure 3). 11 h old males were distributed within the stages 2-4 in the following proportions: 42%, 17% and 42%. After 14 h post-emergence, all males had their genitalia rotated at least 135° (stage 3), and more than 90% of them were already fully rotated. The examination of one-week-old males revealed that all males fully completed the rotation.

Genitalia rotation of the wild-caught specimens started a few hours after emergence as 40% of them were already in the second stage of rotation when 5.5 h old (Table 1). After 12.5 h more than 60% of the males had reached stage 3. The length of stage 3 lasted for ca 10 h as complete rotation was recorded only 23.5 h post-emergence, and this for only 40% of them. Comparison of the rotation degree between laboratory and wild males was made for the shared sampling times between the two male populations, *i.e*. 5.5 h, 12.5 h and 17 h post-emergence. A statistically significant difference was found when comparing the mean rotation stage at 12.5 h (t_5.3 _= 3.48, *P *< 0.01) and 17 h (t_7.3 _= 7.34, *P *< 0.001) (Table 1).

**Table 1:** Stages of terminalia rotation for laboratory and wild males at different time after emergence.

Age (hrs)	Stage 0			Stage 1			Stage 2			Stage 3			Stage 4		
	
	Lab	Wild		Lab	Wild		Lab	Wild		Lab	Wild		Lab	Wild	
5.5	86	60	NS	14	40	NS									
12.5				0	12.5	NS	0	25	*	6.7	62.5	***	93.3	0	***
17							0	25	***	4	75	***	96	0	***

Percentage of laboratory colony (Lab) and males collected on the field (Wild) with the terminalia rotated in the different stages. NS indicates a non significant difference between laboratory and wild males; * and *** indicate a significant difference (*P *< 0.05 and *P *< 0.001 respectively). Sample sizes were 14, 39 and 57 for the laboratory males and 5, 8 and 8 for the wild males at 5.5, 12.5 and 17 h post-emergence respectively.

In this experiment, the frequencies of clockwise and counter-clockwise rotation were not significantly different for each observation point, with mean clockwise and counter-clockwise rotation frequencies of 44.6% and 55.4% respectively. As expected, the control group of virgin females held in a cage without males did not lay any eggs. Female insemination was checked for the groups in which males aged 11 to 18.5 h old. At least one female was found inseminated in all groups; the proportion of spermathecae containing sperm varied from 20% to 40%. Oviposition occurred in all the cages with a low number of eggs laid.

## Discussion

The establishment of disparities between reared and wild insects during colonization can result from selection, genetic drift and inbreeding [23]. Possible methods to avoid and mitigate these in mosquitoes have been discussed but few have been empirically demonstrated to be effective [24]. Several studies reported modifications in the sexual behaviour of long-term laboratory reared insects (house flies [25], screw-worm flies [26,27], bud worms [27] and a shortened sexual maturity period (Mediterranean fruit flies [28]).

Between 2004 and 2009, more than one hundred generations of the Dongola strain have been reared under laboratory conditions, allowing the possibility that selective pressures would lead to purifying selection of particular traits. The present work was prompted by unexpected data obtained during irradiation experiments where the expected sterility levels were not reached. The mating of radio-sterilized males with females separated from males less than 16 h post-emergence never allowed the achievement of full sterility with the five-year-old laboratory colony. A high proportion of females were inseminated at this age as mosquitoes from the laboratory colony were shown to be already sexually competent a few hours after emergence. As early as 11 h post-emergence, males were able to copulate and females were receptive. In spite of a much higher temperature, wild males collected in the field as pupae required twice as much time as laboratory males to complete the rotation of their genitalia. This observation and comparison with previous results suggest a rearing induced selection of the males to sexually mature more rapidly.

Sexual maturity in male mosquitoes is reached after a 180° rotation of the genitalia and the maturation of sexual organs and antennal fibrillae [29]. Approximately 20 h would be required for wild males collected in the field to become sexually mature which is in agreement with the data reported by Mahmood & Reisen [16]. A deceleration in genitalia rotation beyond 90° has been reported for an aedine species [11]; a similar pattern seems to exist in the Dongola wild males, but was not evident in the laboratory males. Provost *et al *[11] reported an increase of the rotation rate with temperature. As the wild males observations had to be conducted at ca 40°C, one could suppose that the completion of this process would be even slower in typical laboratory conditions e.g. 28°C.

Very few females inseminated by 11 to 19.5 h old males laid eggs though spermatheca dissection showed that 20 to 40% of them were inseminated. The low number of ovipositions would suggest that the quantity of sperm actually transferred by males may not always be sufficient to permit oviposition. Indeed, it has been shown with *An. gambiae s.s*. that the oviposition behaviour is triggered by a spermatheca filled with sperm [30], although more recent work demonstrates that the mating plug may alone be sufficient [31]. However, during the mating experiments with sterile males irradiated at 120 Gy, in which females were separated at the adult stage, 6% of them laid fertile progeny (i.e. > 60% fertile eggs) resulting from the early insemination by same age un-irradiated males. This result indicates that those females received enough sperm before the sex-separation process to fertilize and lay viable eggs. Besides, 53% of those females laid egg-batches that were semi-sterile (i.e. >10 and <60% fertile eggs) indicating that fertile then sterile males inseminated them successively and that they used both sperm to fertilize the eggs. The high rate of semi-sterility corroborated the fact that a relatively high proportion of the young emerged males were able to transfer their sperm in the first hours after emergence. It seems likely that most of them inseminated the females only partially thus allowing a subsequent double mating by a sterile male. Mahmood and Reisen [16] suggested that the high rate of multiple matings observed in caged *An. stephensi *and *Anopheles culicifacies *would be induced by an incomplete transfer of sperm, which would mostly occur when the male's reproductive system is partially depleted. They reported the depletion of the accessory glands in newly emerged males or following a mating. However once sexual maturity was reached, the accessory glands were fully filled with secretory cells and male accessory gland fluid. The observation of semi-sterility however suggests that the sperm transferred by young emerged males was already mature, as it has been used to fertilize at least some of the eggs. A possible hypothesis is that the quantity of sperm in the sperm reservoir or the quantity transferred during the mating might be low for the < 18.5 h old males. An alternative explanation could be that newly emerged males were not able to transfer a mating plug after the sperm transfer, and females would eject part of the sperm. Rogers *et al *[31] demonstrated that in anophelines the seminal secretions (mating plug) produced by the male accessory gland and transferred during insemination, promote sperm storage. They suggested that when males failed to transfer the mating plug, females would actively eject the sperm or part of it. It would be of interest to investigate whether the failure of most of the females to oviposit is due to an insufficient quantity of sperm transferred or to the non-transfer of a mating plug.

There are at least five reasons to support the hypothesis of rearing selection pressures for accelerated sexual maturation in males: (*i*) variation in rotation time exists within males of the same age for a given temperature [9]; (*ii*) the observation of males attempting to mate before their genitalia had sufficiently rotated to ensure a successful copulation has been reported for *Ae. aegypti *[9]; (*iii*) the small cages used in laboratory rearing might not allow males to execute a mating swarm and hence favour individual mating attempts [16]; (*iv*) the management of the stock in the laboratory consists of a situation in which there is no overlapping of generations and all males are of a similar age range of approximately three days; (*v*) genetic selection on insects due to rearing pressures have already been reported [32,33]. Such behaviours could favour males that would complete early sexual maturation and this phenomenon would be purified over generations.

The precocious sexual maturity we observed in males may have occurred as well in females. Indeed, they both showed a reduced sexual maturity period as compared with typical values from the literature [15,16]. However the data presented here do not allow us to state whether males and females evolved simultaneously or if one sex was already pre-adapted for early mating. Lima *et al *[34] mentioned the evolution of male *Anopheles albitarsis *mating ability after ca. 10 years of rearing under laboratory conditions, with an improvement of the mating capacity and insemination rates. They suggested that this evolution did not involve females, as the difficulties to mate in a confined space concerned males only. However, Gwadz and Craig [35] reported that females *Ae. aegypti *from four two-year-old laboratory strains showed a significantly shorter refractory period than two young colonized strains, but they did not mention male receptivity. In females *Aedes atropalpus*, Gwadz [36] showed that early receptivity was inherited as it is linked to a juvenile hormone. It is not known however if sexual maturity in males is also moderated by hormones.

Difficulties in establishing colonies of anopheline mosquitoes are often reported and attributed to the incapacity of male swarm formation in a confined space [24]. The adaptation to rearing conditions would necessitate individual mating capacities of the males. Because *Anopheles *mosquitoes' sex ratio is 1:1, it is likely that not all males have an opportunity to mate. Variation among males that may be under selection and that could affect reproductive success include size [37] and the quality of the male accessory gland fluids and sperm [38-40] and genetic factors whose phenotypic effects are unknown [41]. This study showed that, in laboratory settings in which similarly aged males must compete for mates, maturation rate is apparently under selection. As mentioned by Howell & Knols [29], laboratory rearing can lead to a bottleneck because of selective pressures. Negative side effects of such unintentional behavioural selections might present themselves when the reared insects are released in the field and strong differences to their wild counterparts might compromise their survival or mating capacities [42]. Nevertheless the early capacity of mating found in males *An. arabiensis *Dongola might not be a negative attribute in an SIT project, as the released males would be reproductively capable only a few hours after emergence, assuming that they are also able to join or initiate a mating swarm. As part of an SIT program, it is of primary importance that the released sterile males are able to copulate and successfully inseminate wild females as early as possible, so that predation pressures and survival capacities would not have a negative impact on their performance. Indeed, when releasing sterile insects, it is often recommended to wait until adults are sexually mature before their release so that they would be immediately effective in the field. But if a selection of males able to mate shortly after emergence can be induced by the rearing procedure, the possibility of release at the pupal stage becomes advantageous, as the handling can be easier than for adult releases.

## Conclusion

This is the first study describing the temporal process of genitalia rotation for an anopheline species and highlighting the apparent shortening of the sexual maturation period for males of a mosquito species due to rearing conditions. Females were possibly subjected to the same kind of selection but the present study design could not detect it. This result has consequences on laboratory experiments involving virgin females where special care must be taken for the sex-separation process. In addition, rotation of the genitalia is used to determine the age of males collected in the field, using as a reference a correlation between rotation degree, temperature and known age of laboratory reared males [11]. The outcome reported in this study implies that these biological indicators must be used carefully, preferably with a newly colonized strain in order to avoid any discrepancies. Finally, the potential of early mating by freshly emerged males and females of a mass reared strain should be evaluated regularly as it may reflect the evolution and adaptation of the strain in response to laboratory conditions.

## Competing interests

The authors declare that they have no competing interests.

## Authors' contributions

CFO, JG and MQB developed the design of the study. CFO carried out the experimental work, performed the statistical analysis and drafted the manuscript. JG, MQB and GL supervised manuscript preparation and contributed significantly to the final draft of the paper. All authors read and approved the final manuscript.
